# Bridging the regulatory gap: a call for a regional, evidence-informed approach to e-cigarette control in Latin America

**DOI:** 10.3389/fpubh.2025.1689171

**Published:** 2025-09-22

**Authors:** Juan S. Izquierdo-Condoy

**Affiliations:** One Health Research Group, Universidad de las Américas, Quito, Ecuador

**Keywords:** electronic cigarettes, Latin America, public health policy, regulatory frameworks, public health

## Abstract

Electronic cigarettes, a subset of electronic nicotine delivery systems (ENDS), have rapidly gained global popularity, with adolescent and young adult uptake emerging as a key public health concern. Promoted as “healthier alternatives” to smoking and often marketed as cessation aids, these devices have not created the anticipated risk-reduction environment and instead threaten to erode hard-won gains in tobacco control. While international experience highlights the potential effectiveness of policies such as taxation, age restrictions, marketing bans, and smoke-free environment laws, results remain heterogeneous and context-dependent, with enforcement playing a critical role. In Latin America, tobacco use declined from 28% in 2000 to 16.3% in 2020 under the World Health Organization (WHO) Framework Convention on Tobacco Control (FCTC), yet electronic cigarette regulation has lagged, resulting in what can be described as an “unregulated epidemic.” Policy responses remain fragmented: some countries enforce comprehensive bans, others apply partial measures, and many still lack specific frameworks, leaving critical gaps in taxation, surveillance, and digital marketing oversight. This regulatory asymmetry facilitates youth-oriented market expansion and the re-normalization of nicotine consumption. Drawing on global lessons, this perspective argues that Latin America must avoid replicating the decades-long delay experienced with tobacco regulation. Instead, the region requires harmonized, evidence-informed, and context-sensitive frameworks that integrate ENDS-specific measures—such as flavor restrictions, comprehensive advertising bans, nicotine caps, and strict control of online sales—supported by robust enforcement and multisectoral coordination. Such an approach offers the best opportunity to contain this unregulated epidemic, safeguard public health, and protect future generations across the region.

## 1 Introduction

As of 2021, an estimated 68 million people worldwide use electronic cigarettes (also known as e-cigarettes), a subset of electronic nicotine delivery systems (ENDS), with a market comprising hundreds of brands and cartridges containing varying concentrations of nicotine, propylene glycol, and glycerin (1). Among adolescents, current e-cigarette use shows substantial variation across countries, with a pooled prevalence of 9.2% in the past 30 days across 75 countries and rates as high as 33.2% in some settings (2). In the United States, the age-standardized prevalence of current e-cigarette use among adults was 6.9% in 2021, rising to 18% among young adults aged 18–24 years, with nearly three-quarters of users aged 18–20 reporting no prior history of combustible cigarette use (3). Similarly, Global Adult Tobacco Survey (GATS) data show that awareness and experimentation are increasing worldwide, with current use reaching 4.4% in Russia and 2.7% in Costa Rica, and youth prevalence peaking at 10.5% in Russia and 7.6% in Ukraine (4). This rapid expansion has raised global concerns about their potential adverse health effects (5), their conflict with smoke-free laws, and their role as a gateway to the consumption of other harmful substances, particularly among individuals without a history of smoking (6, 7). In Latin America, although sustained efforts under the World Health Organization (WHO)’s Framework Convention on Tobacco Control (FCTC) reduced tobacco use prevalence from 28% in 2000 to 16.3% in 2020 (8), the vaping landscape shifted dramatically: retail sales rose from USD 21 million in 2015 to USD 94.2 million in 2020, with a parallel increase in adolescent and young adult prevalence, a key concern for regulation (9).

Although promoted as “healthier alternatives” and marketed as cessation aids, electronic cigarettes have not produced the anticipated risk-reduction environment (10). The prevailing public health strategy is now to regulate these products with rigor at least equivalent to that applied to conventional cigarettes (11). Since their creation in 2003, commercialization in China in 2004, and subsequent global expansion in 2006, these devices have rapidly gained popularity, including among individuals who had never smoked, thereby increasing the risk of nicotine addiction (6, 12). The initial absence of regulation facilitated unrestricted access among adolescents and school-aged children, accelerating their uptake in younger populations (13–15). This trend threatens to erode hard-won gains in tobacco control across Latin America by re-normalizing nicotine consumption, reversing declines in adolescent smoking, and undermining compliance with smoke-free laws.

The regulation of electronic cigarettes has thus emerged as a complex and evolving issue, with divergent approaches worldwide. The most widely studied strategies include taxation, marketing restrictions, and age limits for purchase (16). However, substantial challenges remain: many countries apply lower tax rates to electronic cigarettes than to conventional tobacco, potentially undermining public health objectives and promoting adolescent use (17). Within this context, Latin America faces the urgent challenge of anticipating risks and adapting global lessons to its unique social, cultural, and epidemiological realities, thereby avoiding the decades-long trajectory required for tobacco control. It is also important to highlight that e-cigarettes are not explicitly covered under the WHO FCTC. Countries often rely on interpretations, and several COP decisions invite the regulation of ENDS; nevertheless, this legal ambiguity complicates enforcement and underscores the need for new, dedicated e-cigarette policies.

The objective of this perspective article is to provide a critical analysis of regulatory gaps in Latin America, highlight emerging risks associated with electronic cigarettes, and propose strategic axes for advancing toward a comprehensive and context-sensitive regulatory framework.

## 2 The unregulated epidemic: gaps in Latin American policy

Despite significant advances in tobacco control over recent decades, Latin America now faces a paradoxical and escalating challenge with electronic cigarettes and other ENDS. Unlike conventional tobacco, the scientific evidence guiding policymaking in the region remains limited, as most studies and regulatory experiences originate from North America and Europe (16). This external dependency constrains the capacity of local governments and fosters critical knowledge gaps precisely as the use of these devices expands rapidly, particularly among adolescents, young adults, and increasingly among new users with no history of tobacco consumption (18, 19). Although 96% of the regional population is currently protected by at least one of the six WHO-recommended tobacco control measures (20), this momentum has not extended to electronic cigarettes, generating what can be described as an “unregulated epidemic” with profound health and social implications.

Policy responses across Latin American nations are fragmented and fall into three main models: total prohibition, partial regulation, and regulatory inaction. For clarity, I summarized these categories—derived from WHO FCTC reports and regional legal frameworks—in comparative tables rather than long country lists in the main text (Tables 1–3). Eight countries—including Argentina, Brazil, Mexico, Uruguay, and Venezuela—have implemented complete bans on sales, imports, and promotion, extending restrictions to public use and advertising, with several of these provisions reaffirmed or updated during the late 2020s (21–25) (Table 1). In contrast, 13 countries such as Chile, Colombia, Costa Rica, Ecuador, Paraguay, and Peru have adopted partial measures, while several others still lack specific frameworks. These include restrictions on sales to individuals under 18, prohibitions on use in smoke-free environments, and advertising limitations, but with substantial gaps in critical areas such as taxation and health product registration (21, 26, 27) (Table 2). Meanwhile, 14 states—including the Dominican Republic, Guatemala, Honduras, El Salvador, and Bolivia—still lack specific regulatory frameworks, leaving commercialization virtually unrestricted and facilitating market penetration into vulnerable populations (28) (Table 3). This regulatory heterogeneity is summarized across Tables 1–3, which compare current legal measures across the region.

**Table 1:** Electronic cigarette regulatory policies in Latin America countries with total bans.

Country	Legal framework	Year	Sales	Importation	Manufacturing	Storage	Personal use	Use in public spaces	Advertising
Argentina	ANMAT Disp. No. 3226/2011; Law 26.687	2011	Prohibited	Prohibited	Not specified	Not specified	Not specified	Prohibited in smoke-free spaces	Completely prohibited
Brazil	ANVISA RDC No. 46/2009; RDC No. 855/2024	2009/2024	Prohibited	Prohibited	Prohibited	Prohibited	Not specified	Prohibited in enclosed collective venues	Prohibited since 2009
Mexico	General Tobacco Control Law; Decrees 2021–2022	2021–2022	Prohibited	Prohibited since 2021	Not specified	Not specified	Not specified	Prohibited in 100% smoke-free spaces	Completely prohibited
Nicaragua	Resolution 334–2021; Circular CT/116/2022	2021–2022	Prohibited	Prohibited (including personal use)	Not specified	Not specified	Prohibited	Completely prohibited	Implicitly prohibited
Panama	Decree 1838/2014; Law 315/2022 (annulled); Res. 146/2025	2014–2025	Variable depending on period	Administrative control	Not specified	Not specified	Not specified	Prohibited in public spaces	Prohibited
Suriname	Updated Tobacco Control Law	2023	Prohibited	Prohibited	Prohibited	Not specified	Not specified	Not specified	Prohibited
Uruguay	Decree No. 534/009; Decree No. 302/017	2009/2017	Prohibited since 2009	Prohibited	Prohibited	Not specified	Not specified	Prohibited since 2017	Prohibited since 2009
Venezuela	Resolution 090–2023	2023	Prohibited	Prohibited	Prohibited	Not specified	Prohibited	Completely prohibited	Completely prohibited

**Table 2:** Electronic cigarette regulatory policies in Latin America countries with partial regulations.

Country	Legal framework	Year	Minimum age	Public spaces	Advertising	Taxes	Labeling	Observations
Chile	Law 21.642	2025	18 years	Prohibited (equated to tobacco)	Prohibited	Not specified	Mandatory health warnings	Geographic restriction: 100 m from schools
Colombia	Law 2,354	2024	18 years	Prohibited in enclosed spaces	Completely prohibited	Not specified	Graphic warnings (1-year deadline)	Regulatory equivalence with tobacco
Costa Rica	Law No. 10066	2022	18 years	Prohibited (specific listing)	Heavily restricted	ISC approx. 20%	Mandatory health registration	Nicotine limit 2%; synthetic nicotine prohibited
Ecuador	LORCT 2011; in force since 2018	2018	18 years	Prohibited in enclosed spaces	Prohibited	Not specified	Graphic warnings for nicotine products	Mandatory ARCSA registration; flavor debate ongoing
Paraguay	Law enacted June 2025 (pending enforcement)	2025	18 years	Prohibited in enclosed spaces	Completely prohibited	ISC 22–24%	To be defined	Sales only where tobacco is sold; 2% nicotine limit
Peru	Law No. 32159	2024	18 years	Prohibited (enclosed public spaces)	Completely prohibited	Not specified	Health warnings	Prohibits youth-targeted flavorings

**Table 3:** Electronic cigarette regulatory policies in Latin America countries without specific or minimal regulation.

Country	Current situation	Projects/initiatives	Legal sale	Existing restrictions
Dominican Republic	No specific law	Senatorial project 2023–2024 (returned to committee)	Yes, without restrictions	Analogy with Law 48–00 (tobacco control)
Guatemala	No specific regulation	Expressions of health concern	Yes	Tobacco control law does not include e-cigarettes
Honduras	No specific regulation	Not reported	Yes	Tobacco control law does not include e-cigarettes
El Salvador	No specific regulation	Not reported	Yes	Weak tobacco control regulation
Bolivia	No specific regulation	Not reported	Yes (limited)	Tobacco Control Law 2015 does not include e-cigarettes

Comparative analysis shows that most governments have largely replicated tobacco control policies without developing targeted responses for electronic cigarettes. While minimum age restrictions are broadly defined, other measures have been poorly or only marginally implemented. A notable example is taxation, currently observed only in Costa Rica (20%), Paraguay (22–24%), and the Dominican Republic (20%, under proposal) (29–31). Although recent reforms in several countries suggest a gradual trend toward stricter regulation, in practice many governments continue to treat electronic cigarette regulation as a mere extension of tobacco control. By “mere extension,” I refer to policies that replicate traditional tobacco control measures—such as taxation, age restrictions, and smoke-free laws—without adapting them to ENDS-specific challenges. These include regulating nicotine concentration, restricting device modifications, addressing flavoring diversity, and controlling digital marketing. Without these targeted provisions, tobacco-based policies alone are insufficient to contain ENDS proliferation. This approach is inadequate given the distinct implications of these devices, marketed as cessation tools but increasingly consumed by youth and first-time users, with long-term harms still insufficiently understood. Although some countries—such as Uruguay and Brazil—have positioned themselves as pioneers with comprehensive bans, these represent exceptions rather than a regional trend (23, 24).

The result is a regulatory asymmetry that undermines hard-won gains in tobacco control and enables the consolidation of a new market that, under the guise of a cessation tool, has become progressively normalized among younger populations. A harmonized framework for the region would entail coordinated minimum standards—comprehensive advertising bans, restrictions on youth-targeted flavors, nicotine concentration limits, regulation of online sales, and consistent enforcement—supported by regional organizations such as Pan America Health Association (PAHO) to reduce policy asymmetry across the 33 Latin American countries.

## 3 Learning from global experiences

International experience provides valuable lessons on the effectiveness of regulatory measures to control electronic cigarette use, although outcomes are often heterogeneous and context-dependent. The most frequently evaluated policies include taxation, age restrictions, marketing limitations, and smoke-free environment laws.

Regarding taxation, evidence suggests a generally favorable, though variable, impact on reducing consumption, particularly among adolescents. In South Korea, for instance, an increase in the local tax from USD 0.40 to 1.60 per milliliter was associated with a decline in adolescent prevalence from 4.7% to 4.0% (32). Other studies have confirmed reductions in use among both youth and adults (33, 34), whereas analyses in the United States found no significant associations, likely due to low tax rates or the ease of alternative Access (35). Taxes appear to be effective when substantial and accompanied by mechanisms that limit informal markets. These findings highlight that age restrictions alone may be insufficient without robust fiscal control.

Age restrictions—particularly “Tobacco 21” laws—show more consistent effects in reducing youth prevalence when enforced effectively (33, 36). Nevertheless, mixed results have also been reported: some studies noted compensatory increases in conventional cigarette use (37) or reliance on alternative acquisition channels, such as proxy purchases (34).

Marketing restrictions demonstrate that comprehensive bans on advertising, health claims, and appealing packaging are associated with lower exposure and reduced use among adolescents and young adults (32, 38). However, systematic reviews have noted that effectiveness may be limited if measures are not comprehensive or lack proper enforcement (16). Additionally, some studies suggest that less restrictive environments may have differential effects, supporting cessation in adults while simultaneously stimulating initiation among youth (15).

Smoke-free environment laws have shown cumulative benefits when combined with taxation and age restrictions, contributing to the denormalization of consumption (34). This mirrors the experience with traditional tobacco control, where the WHO’s comprehensive MPOWER strategy reduced smoking prevalence in the Americas from 28% in 2000 to 16.3% in 2020 (39).

Beyond these traditional measures, several e-cigarette-specific regulations provide further lessons: the European Union’s Tobacco Products Directive sets nicotine concentration limits and standardized packaging requirements (40); New Zealand employs a health product licensing model for nicotine-containing devices; and Canada restricts flavors attractive to youth (41, 42). These examples underscore that regulation cannot simply mirror tobacco control but must be tailored to ENDS’ unique features.

Overall, international evidence underscores that no single measure is sufficient. The most effective policies are those combining multiple approaches simultaneously—substantial taxes, strict age limits, comprehensive marketing bans, and 100% smoke-free environments—adapted to local contexts and reinforced by effective enforcement. The global lesson is that effective regulation integrates traditional tobacco strategies with ENDS-specific measures targeting product design, nicotine content, flavors, and digital marketing. For Latin America, this evidence represents a crucial starting point for transitioning from partial or inactive frameworks toward integrated strategies, thereby avoiding with electronic cigarettes the protracted learning curve already experienced with tobacco.

## 4 A call for a regional, evidence-informed approach

Global advances in electronic cigarette regulation provide useful lessons but also demonstrate that Latin America cannot simply import foreign models without adapting them to its own social, cultural, and epidemiological contexts. In 2023, the WHO issued a global call to action on e-cigarettes, urging countries to adopt baseline measures—including flavor bans, plain packaging, nicotine concentration caps, advertising restrictions, and regulation of online sales—while allowing adaptation to local realities (43). The evidence reviewed highlights that measures such as taxation, age restrictions, marketing limitations, and smoke-free environment policies have positive effects, though heterogeneous and dependent on the level of enforcement (16, 32, 33, 38). However, experiences in countries such as Mexico—where formal bans exist but youth consumption continues to rise (19), illustrate that regulatory design alone is insufficient without complementary strategies for enforcement, education, and control of digital advertising.

The Latin American situation is particularly challenging. Although pooled data suggest an adolescent prevalence of electronic cigarette use of 18.9%, this figure is likely underestimated due to the scarcity of studies and the limited epidemiological surveillance systems in the region (19, 44). Moreover, key associated factors include concurrent use of tobacco and other substances, peer influence, and exposure to social media advertising (19). These challenges are exacerbated in Latin America by weak regulation of digital promotion, limited enforcement capacity, porous borders that facilitate informal trade, and fragmented surveillance systems (45).

International evidence also shows that the integration of multiple policies reinforces the cumulative effects of each measure. For Latin America, advancing a regional strategy therefore requires combining approaches: flavor and marketing restrictions, particularly on social media; visible labeling with health warnings; strict regulation of physical and online points of sale; and incorporation into tobacco cessation strategies (19). Additionally, innovative measures such as reclassifying nicotine-containing vaping products as consumer goods regulated under consumer protection agencies could help close regulatory gaps and curb informal trade. If ENDS are regulated as consumer products, the WHO recommends a set of minimum standards: nicotine concentration caps, licensing of vendors, plain packaging, and bans on youth-oriented flavorings (43). In Latin America, implementing this model would require specific strategies to control informal markets and strengthen institutional enforcement capacity.

In this context, a call to action is warranted for all stakeholders involved in electronic cigarette use. Policymakers and decision-makers must implement comprehensive, coherent regulatory frameworks that are evidence-based yet tailored to Latin American social and cultural realities, strengthening enforcement and avoiding fragmented or merely reactive approaches. Academia and the scientific community should expand epidemiological, market, and health impact research, with particular emphasis on youth consumption patterns and long-term adverse effects. The education sector should incorporate preventive and health literacy content into school and university curricula to raise adolescent awareness of vaping risks. Digital platforms and advertising regulators must establish strict oversight mechanisms to limit covert promotion, regulate online sales, and reduce youth exposure to pro-vaping messages. Only through a multisectoral approach will it be possible to contain this unregulated epidemic in the region.

A regional response must also account for the role of the e-cigarette industry. Like the historical tactics of the tobacco sector, companies employ aggressive digital marketing, introduce youth-oriented flavors, and lobby against restrictive laws. These strategies accelerate market penetration and weaken enforcement, particularly in countries with fragile regulatory systems (46, 47). Therefore, comprehensive regulation must include strict oversight of industry practices, transparency in lobbying, and restrictions on corporate marketing to prevent public health gains from being undermined.

Finally, while some countries such as England and New Zealand promote ENDS as part of cessation strategies, in Latin America their predominant use is recreational and concentrated among youth, often associated with alcohol and other substance use (15). Latin America has the opportunity to anticipate: a regional approach that is evidence-informed yet sensitive to local realities can prevent the region from repeating the decades-long cycle of struggle against tobacco. This paradigm shift, inspired by the WHO’s MPOWER policies, will not only protect public health but also help reduce social inequities and improve human development indicators across the region (39, 48).

## 5 Conclusion

The regulation of electronic cigarettes in Latin America stands at a critical crossroads. While some countries have advanced with comprehensive bans and others with partial measures, most show persistent regulatory gaps that enable youth-oriented market expansion and the re-normalization of nicotine use. Although international experience shows that no single measure or policy has proven fully effective on its own, the regional context underscores the need not to import external models, but rather to anticipate, design, and implement a harmonized framework that combines traditional tobacco measures with ENDS-specific provisions—such as comprehensive advertising bans, flavor restrictions, nicotine caps, online sales regulation, health warnings, and vendor licensing—tailored to the region’s social, cultural, and epidemiological realities. Only through a multisectoral approach that also addresses industry tactics (marketing, lobbying, and product design) and aligns public health, education, consumer protection, and digital oversight will it be possible to contain this “unregulated epidemic” and protect future generations while safeguarding hard-won gains in tobacco control, with tangible benefits for equity and human development across Latin America.

## Data Availability

The original contributions presented in the study are included in the article/supplementary material, further inquiries can be directed to the corresponding author.
